# Safety and immunogenicity of a Vi-DT typhoid conjugate vaccine: Phase I trial in Healthy Filipino adults and children

**DOI:** 10.1016/j.vaccine.2018.05.038

**Published:** 2018-06-18

**Authors:** Maria Rosario Capeding, Samuel Teshome, Tarun Saluja, Khalid Ali Syed, Deok Ryun Kim, Ju Yeon Park, Jae Seung Yang, Yang Hee Kim, Jiwook Park, Sue-Kyoung Jo, Yun Chon, Sudeep Kothari, Seon-Young Yang, Dong Soo Ham, Ji Hwa Ryu, Hee-Seong Hwang, Ju-Hwan Mun, Julia A. Lynch, Jerome H. Kim, Hun Kim, Jean-Louis Excler, Sushant Sahastrabuddhe

**Affiliations:** a Research Institute for Tropical Medicine, Manila, Philippines; b International Vaccine Institute, Seoul, Republic of Korea; c SK Chemicals, Seoul, Republic of Korea

**Keywords:** Typhoid, Conjugate vaccine, Vi-DT, Safety, Immunogenicity, The Philippines

## Abstract

**Background:**

Typhoid fever remains a major public health problem in low- and middle-income countries where children aged 2–14 years bear the greatest burden. Vi polysaccharide is poorly immunogenic in children <2 years of age, and protection in adults is modest. The limitations of Vi polysaccharide vaccines can be overcome by conjugation of the Vi to a carrier protein. A typhoid conjugate vaccine composed of Vi polysaccharide conjugated to diphtheria toxoid (Vi-DT) has been developed. The Phase I study results are presented here.

**Methods:**

This was a randomized, observer-blinded Phase I study to assess the safety and immunogenicity of Vi-DT compared to Vi polysaccharide vaccine, conducted in Manila, Philippines. Participants enrolled in an age de-escalation manner (18–45, 6–17 and 2–5 years) were randomized between Test (Vi-DT, 25 µg) administered at 0 and 4 weeks and Comparator (Vi polysaccharide, Typhim Vi® and Vaxigrip®, Sanofi Pasteur) vaccines.

**Results:**

A total of 144 participants were enrolled (48 by age strata, 24 in Test and Comparator groups each). No serious adverse event was reported in either group. Solicited and unsolicited adverse events were mild or moderate in both groups with the exception of a 4-year old girl in Test group with grade 3 fever which resolved without sequelae. All participants in Test group seroconverted after first and second doses of Vi-DT while the proportions in the Comparator group were 97.1% and 97.2%, after first dose of Typhim Vi® and second dose of Vaxigrip®, respectively. Vi-DT showed 4-fold higher Geometric Mean Titers (GMT) compared to Typhim Vi® (adjusted for age strata, p < 0.001). No further increase of GMT was detected after the second dose of Vi-DT. Anti-DT IgG seroresponse rates were 81.2% and 84.5% post first and second Vi-DT doses, respectively.

**Conclusions:**

Vi-DT vaccine was safe, well-tolerated and immunogenic in participants aged 2–45 years.

ClinicalTrials.gov registration number: NCT02645032.

## Highlights

• Vi-DT is safe and well tolerated in participants aged 2–45 years.
• Significantly higher immune responses post Vi-DT compared to Vi polysaccharide vaccine.
• No further increase in GMT post second dose as compared to post first dose of Vi-DT.

## Nomenclature

**Abbreviations**
AE: adverse event
DT: diphtheria toxoid
GCP: good clinical practice
GMT: geometric mean titers
GMF: geometric mean fold
IRB: institutional review board
IVI: International Vaccine Institute
LMIC: low- and middle-income countries
PFDA: Philippines Food and Drug Administration
PP: per protocol
RITM: Research Institute for Tropical Medicine
SAE: serious adverse event
SBA: serum bactericidal assay
SMC: Safety Monitoring Committee
Vi: *Salmonella* typhi capsular polysaccharide
Vi-DT: diphtheria toxoid conjugated Vi-polysaccharide vaccine
Vi-PS: *Salmonella* typhi capsular polysaccharide vaccine

## 1 Introduction

Typhoid fever is one of the most common causes of bacteremia in several low- and middle-income countries (LMIC) and has been estimated to cause 11–21 million cases and 145,000–161,000 deaths per year [1]. Symptoms include fever, abdominal pain, and nausea, which last between one to four weeks, and 1–2% of hospitalized cases result in death [2], [3]. Improved sanitation contributed to the sharp decline of typhoid fever in industrialized countries during the early 20th century [4], [5] but such infrastructure is slow to materialize in places where the disease remains endemic [4], [6]. Vaccination may provide a short-to-medium term measure to abate the typhoid burden of disease [2]. It is therefore essential to consider a comprehensive approach that combines targeted vaccination of at-risk populations as a short- to medium-term prevention measure, along with longer term solutions of improvements of water and sanitation and living standards [7].

Several safe and effective typhoid vaccines that could help reduce disease burden are licensed and available. Three or four doses of orally administered live-attenuated Ty21a provide about 50–70% protection for at least 7 years and is licensed in capsule form from 5 years of age or as a liquid formulation from 2 years of age, although the liquid formulation is not commercially available [8], [9], [10]. The single-dose injectable Vi polysaccharide vaccine provides similar levels of protection for at least 3 years and is licensed from 2 years of age [11], [12]. Although Vi polysaccharide vaccination has been shown to protect individuals from typhoid fever, it has several limitations due to T cell-independent properties. Immune responses to bacterial capsular polysaccharides are characterized by T-cell independence, lack of affinity maturation, poor antibody subclass switching and inability to generate memory. This limits their use in children less than two years of age [13], [14]. These limitations can be overcome by conjugation of the Vi polysaccharide to a carrier protein. Conjugation of the polysaccharide to a carrier protein converts the immune response to T-cell dependent characterized by affinity maturation, subclass switching and induction of memory [15]. Two Vi polysaccharide vaccines conjugated to tetanus toxoid as carrier protein are licensed in India for use from 3 to 6 months of age [16]. The immunogenicity of typhoid conjugate vaccines in children under 2 years of age is an important advance, [17] given the significant burden of disease in young children and infants [18], [19].

The International Vaccine Institute (IVI, Seoul, Republic of Korea) developed a typhoid conjugate vaccine (Vi-DT) where the Vi polysaccharide (a clinical isolate from India (C6524)) is conjugated to diphtheria toxoid as carrier protein. In order to meet the global demand of typhoid conjugate vaccines, IVI has transferred this technology to SK Chemicals, Republic of Korea for future commercialization.

## 2 Materials and methods

The clinical study (Clinicaltrials.gov NCT02645032) was approved by the Philippines Food and Drug Administration (PFDA) and the Institutional Review Boards (IRB) of the Research Institute for Tropical Medicine (RITM) and IVI. The study was conducted in accordance with the ICH E8 Guidelines for Good Clinical Practice (GCP) and the ethical principles of the Declaration of Helsinki. Before any study intervention, a written informed consent was obtained from eligible adult participants and from the parents or legal guardians of participants aged 2–17 years. Assent was also obtained from 7 to 17 years old children according to the 2011 Philippines National Ethics Guidelines.

### 2.1 Study design and participants

This was a randomized, observer-blinded, Phase I study to assess the safety and immunogenicity of 25 µg Vi-DT typhoid conjugate vaccine (Test vaccine) compared with Vi polysaccharide typhoid vaccine (25 µg) (Typhim Vi®, Sanofi Pasteur) (Comparator vaccine). Since Test and Comparator vaccines differ in their presentation, the study was observer-blinded (safety evaluators and other trial staff remained blinded with the exception of the vaccine administrator) to ensure evaluator’s blinding to prevent bias in assessment of adverse events. This study was conducted at RITM, Manila, the Philippines, from May 2016 to Feb 2017. Healthy Filipino participants aged 2–45 years were enrolled into 3 cohorts of 18–45, 6–17, and 2–5 years in an age de-escalation manner.

The primary objective of the study was to evaluate the safety of Vi-DT, while the secondary objectives were to assess the immunogenicity of Vi-DT comparatively to Typhim Vi®.

### 2.2 Vaccines

The Test vaccine (Vi-DT) used in this study is a purified Vi polysaccharide conjugated to diphtheria toxoid. The vaccine containing two active ingredients, 25 µg of purified Vi polysaccharide (*S.* Typhi C6524) and diphtheria toxoid (*Corynebacterium diphtheria* PW No.8) formulated with stabilizers was administered intramuscularly as 0.5 mL/vial. The Comparator vaccine Typhim Vi® (Sanofi Pasteur) contained 25 µg of purified Vi polysaccharide (*S.* Typhi Ty2). Since the Comparator vaccine is administered as a single dose, the second dose administered was a flu vaccine (Vaxigrip®, split viron, inactivated influenza vaccine, southern hemisphere, Sanofi Pasteur) to keep the blinding. Both Comparator vaccines were given intramuscularly in the deltoid muscle of the left upper arm for participants aged ≥3 years and in the anterolateral left thigh *vastus lateralis* muscle for children aged 2–3 years. Vaccines were stored at +2–8 °C. The first and second doses of Test and Comparator vaccines were administered 4 weeks apart.

### 2.3 Assessment of safety and reactogenicity

Participants were assessed for immediate reactions up to 60 min following vaccination. Participants/parents/guardians were provided with a thermometer and diary cards (DC) to record axillary temperature and any adverse event (AE) daily up to 7 days after each dose for solicited or up to 28 days for unsolicited adverse events. Local reactogenicity events (at the site of injection) included pain, tenderness, erythema/redness, swelling/induration and pruritus after study vaccine administration. Tenderness as a solicited reaction was not sought in children as it is difficult to assess in younger participants. Tenderness was sought in adults and adolescents only. Solicited systemic AEs included fever, headache, fatigue, arthralgia, myalgia, chills, nausea/vomiting and acute allergic reaction after study vaccine administration. Unsolicited Adverse Events were defined as, any other adverse event that occurred from the date of administration of the investigational product (IP) to 28 days following each dose (Days 0–28). Unsolicited AEs were classified into System Organ Class (SOC) and Preferred Term (PT) using MedDRA (version 18.1, 2015). Participants were invited to visit the study site on Days 3, 7 and 28 after each vaccine dose for safety assessment and in case of development of any AE. Serious adverse events (SAE) were recorded for the entire duration of the study. Abnormal lab values (hematology and biochemistry) were graded using NIAID toxicity grading criteria [20]. In the age de-escalating procedure, blinded safety data of the older cohort was reviewed by an independent Safety Monitoring Committee (SMC). Upon SMC recommendation, RITM and IVI IRBs approval were required before proceeding with lower age group enrolment.

### 2.4 Assessment of immunogenicity

Blood samples were collected from all trial participants prior to vaccination and 28 days post first and second doses. Sera were stored at -80 °C to -20 °C until analysis.

Anti-Vi IgG in sera were measured by ELISA as previously described [21] with the exception of added pre-coating of poly-L-lysine (1 μg/well) (Sigma, USA) prior to Vi coating (0.2 μg/well) onto microplates. A Vi-reference serum (Vi IgG_R1,2011_) was used and anti-Vi IgG levels were expressed in μg/mL [21]. Anti-DT IgG levels were measured by ELISA (Diphtheria IgG ELISA, IBL International GmbH, Hamburg, Germany). Serum bactericidal assay (SBA) was performed using a semi-automated assay as previously described [22]. Lower limit of detection for anti-Vi IgG, anti-DT IgG, and SBA is 0.003 μg/ml (internal qualification, unpublished data), 0.004 IU/ml (manufacturer’s instruction), 10 (reciprocal serum-dilution fold inhibiting 50% of bacterial growth), respectively. For statistical analysis, antibody levels including anti-Vi IgG, anti-DT IgG, and SBA titers below level of detection were assigned as half of the detectable value.

### 2.5 Statistical analysis

Since the primary endpoint is safety, the overall study sample size was calculated so that for each vaccine and age cohort, with 24 participants per vaccine and age group, there is a 90% probability of observing at least one participant with an adverse event if the true rate of such an event is 10%.

Eligible participants in each age cohort were randomly assigned to receive either Vi-DT or Comparator vaccine in a 1:1 ratio. Three randomization lists, one for each age cohort were generated by a statistician independent of the study at IVI. The randomization lists contained sequential numbers unique to each participant in each age group. Block randomization was employed to ensure an effective balance between interventions within each age cohort.

All randomized participants were included in the analysis of demographics and baseline characteristics. In the safety analysis set, safety was analyzed for all randomized participants who received at least one dose of study vaccines. The immunogenicity analysis was performed in immunogenicity and per-protocol (PP) sets. The immunogenicity set was defined as participants who were randomized, received at least one dose of study vaccine and provided at least one post baseline measure for immunogenicity. The PP analysis set was a subset of the intention-to treat population with no emergent deviation (defined as major deviation from the protocol compromising the safety of the participants and/or the scientific integrity of the protocol), compliance to study procedures, completion of all scheduled visits, and administration of the correct vaccinations.

Demographic characteristics of continuous variables were summarized by number of participants, mean, standard deviation, median, minimum and maximum, and categorical variables were summarized by frequency and percentage in each vaccine group.

The proportion of participants who experienced solicited and unsolicited AEs after each vaccination was provided with the 95% CI. The comparison of proportions for all ages was performed using stratified Chi-square (Cochran-Mantel-Haenszel) test stratified by age, and the comparison in each age cohort was performed using Pearson’s Chi-square test or Fisher’s Exact test.

The proportion of participants with seroconversion (defined as fourfold rise in anti-Vi antibody titers compared to baseline), 4 weeks post first and second doses of Vi-DT and post one dose of Vi polysaccharide vaccine was provided and comparison was performed by stratified Chi-square test (Cochran-Mantel-Haenszel test) for overall age group. Comparison of Geometric Mean Titer (GMT) of antibody response and 95% CI for all ages was performed using analysis of covariance (ANCOVA) adjusted for age cohort. All analyses were performed using SAS 9.4 (SAS Institute, Cary NC). The primary objective of the study was assessment of safety and the study was not powered to show non-inferiority of immunogenicity between Test and Comparator vaccines.

## 3 Results

### 3.1 Study population

**Fig. 1:**
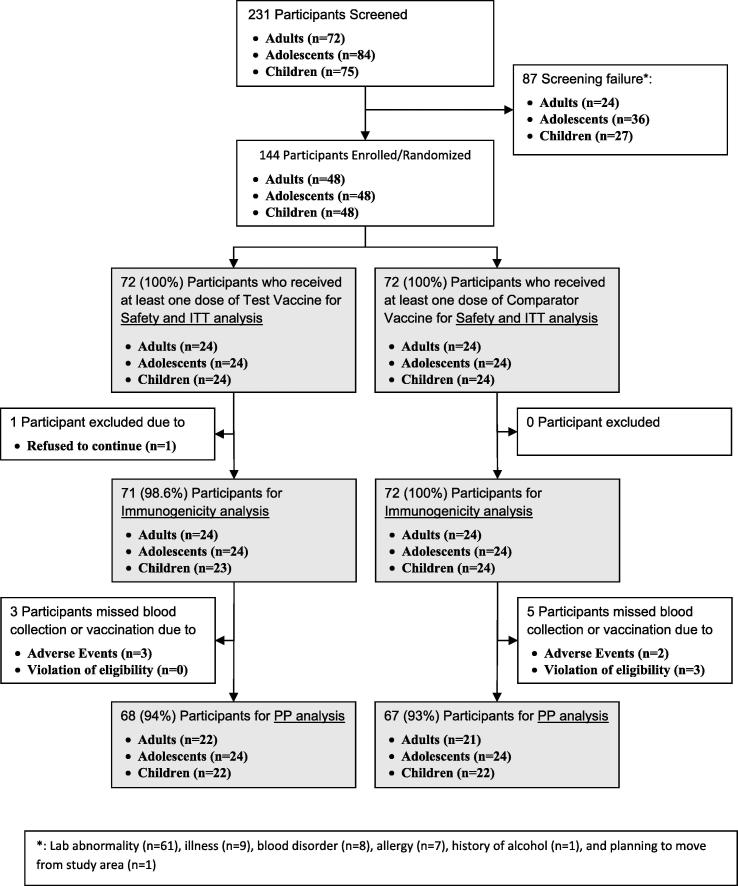
Flow diagram of participant disposition (CONSORT flow diagram) by analysis sets.

Among 231 subjects screened, 144 participants were enrolled and randomized to either Test or Comparator vaccine groups. Of those randomized, 68 (94%) in Test and 67 (93%) in Comparator groups completed the study per protocol (Fig. 1). Both groups were comparable with regard to demographic and baseline characteristics (Table 1).

**Table 1:** Demographic characteristics of the study participants.

Characteristics		Test Group	Comparator Group
Overall		N = 72	N = 72
Gender	Male (%)	46 (63.9)	49 (68.1)
	Female (%)	26 (36.1)	23 (31.9)
Age (years)	Mean (SD)	13.67 (10.0)	14.54 (12.2)
	Median (min, max)	12.00 (2.00, 45.00)	9.50 (2.00, 45.00)

Adults		N = 24	N = 24
Gender	Male (%)	14 (58.3)	16 (66.7)
	Female (%)	10 (41.7)	8 (33.3)
Age (years)	Mean (SD)	25.71 (6.4)	29.63 (8.2)
	Median (min, max)	24 (18, 5)	29 (18, 45)

Adolescents		N = 24	N = 24
Gender	Male (%)	16 (66.7)	15 (62.5)
	Female (%)	8 (33.3)	9 (37.5)
Age (years)	Mean (SD)	11.58 (3.0)	10.54 (3.1)
	Median (min, max)	12 (7, 16)	9.5 (6, 16)

Children		N = 24	N = 24
Gender	Male (%)	15 (62.5)	19 (79.2)
	Female (%)	9 (37.5)	5 (20.8)
Age (years)	Mean (SD)	3.63 (1.0)	3.54 (1.3)
	Median (min, max)	4 (2, 5)	3.5 (2, 5)

### 3.2 Safety evaluation

A total of 22 participants reported immediate reactions in both Test and Comparator groups combined. All age cohorts were comparable for immediate reactions in both groups and reactions were mild. Pain at injection site was the most common immediate reaction reported in both groups with 7% and 15.3% in Test and Comparator vaccine groups, respectively. Tenderness was not elicited in young children but was a common immediate reaction in adolescents with 4.2% and 16.7% in Test and Comparator vaccine groups, respectively.

Table 2 provides the proportion of participants with solicited AEs per vaccine group, all ages combined. The proportion of participants reporting solicited AEs within 7 days after any dose of Test or Comparator vaccine was 38.9% (95% CI: 28.5, 50.4) and 40.3% (95% CI: 29.7, 51.8), respectively. Irrespective of the age and dose, pain and tenderness were the most common solicited AEs reported in both groups. All AEs reported were of mild to moderate severity in all age cohorts from both vaccine groups except a 4-year old girl with grade 3 fever in Test group which resolved without sequelae. Pain, tenderness and headache were the most common solicited AEs reported among adults in both groups. Pain-tenderness and pain-fever were the most common solicited AEs in adolescents and children, respectively.

**Table 2:** Proportion of participants with solicited adverse events per vaccine groups, all ages combined.

	Test Group (N = 72)	Comparator Group (N = 72)
Within 7 days after any dose	Number of participants (%)	Number of participants (%)
All ages	28 (38.9%)	29 (40.3%)
Pain	15 (20.8%)	18 (25.0%)
Tenderness	13 (18.1%)	15 (20.8%)
Redness/Erythema	1 (1.4%)	0 (0.0%)
Swelling Induration	2 (2.8%)	0 (0.0%)
Pruritus associated with injection	3 (4.2%)	0 (0.0%)
Fever	4 (5.6%)	6 (8.3%)
Headache	10 (13.9%)	4 (5.6%)
Fatigue	2 (2.8%)	1 (1.4%)
Muscle ache or myalgia	3 (4.2%)	3 (4.2%)
Nausea/Vomiting	2 (2.8%)	2 (2.8%)
Joint pain or Arthralgia	0 (0.0%)	1 (1.4%)
Chills	0 (0.0%)	1 (1.4%)
Acute allergic reaction	0 (0.0%)	0 (0.0%)

Throughout the study period, unsolicited AEs were reported by 55.6% (95% CI: 44.1, 66.5) and 54.2% (95% CI: 42.7, 65.2) of participants in the Test and Comparator groups, respectively. All unsolicited AEs reported were of mild to moderate severity only and most of them were assessed unrelated to vaccine (Table 3).

**Table 3:** Frequency of solicited and unsolicited adverse events for the entire study period in the Test and Comparator groups for all age groups combined.

Entire study period (day 0 to day 56)		Test Group (N = 72)		Comparator Group (N = 72)	
		Number of AEs	Number of Participants (%)	Number of AEs	Number of Participants (%)

**Solicited AE** (within 7 days after each vaccination)		**67**	**28 (38.9%)**	**64**	**29 (40.3%)**

Severity:	Grade 1 (Mild)	63	28 (38.9%)	57	28 (38.9%)
	Grade 2 (Moderate)	3	3 (4.2%)	7	4 (5.6%)
	Grade 3 (Severe)	1	1 (1.4%)	0	0 (0.0%)
	Grade 4 (Life-threatening)	0	0 (0.0%)	0	0 (0.0%)

Relatedness*:	A1	50	24 (33.3%)	53	26 (36.1%)
	A2	0	0 (0.0%)	0	0 (0.0%)
	A3	0	0 (0.0%)	0	0 (0.0%)
	A4	0	0 (0.0%)	0	0 (0.0%)
	B1	7	3 (4.2%)	1	1 (1.4%)
	B2	2	2 (2.8%)	6	1 (1.4%)
	C	8	6 (8.3%)	4	4 (5.6%)
	D	0	0 (0.0%)	0	0 (0.0%)

**Unsolicited AE**		**61**	**40 (55.6%)**	**63**	**39 (54.2%)**

Severity:	Grade 1 (Mild)	58	38 (52.8%)	60	38 (52.8%)
	Grade 2 (Moderate)	3	3 (4.2%)	3	3 (4.2%)
	Grade 3 (Severe)	0	0 (0.0%)	0	0 (0.0%)
	Grade 4 (Life-threatening)	0	0 (0.0%)	0	0 (0.0%)

Relatedness*:	A1	1	1 (1.4%)	2	2 (2.8%)
	A2	0	0 (0.0%)	0	0 (0.0%)
	A3	0	0 (0.0%)	0	0 (0.0%)
	A4	0	0 (0.0%)	0	0 (0.0%)
	B1	2	2 (2.8%)	0	0 (0.0%)
	B2	5	5 (6.9%)	9	9 (12.5%)
	C	53	33 (45.8%)	52	32 (44.4%)
	D	0	0 (0.0%)	0	0 (0.00%)

**SAE**		**0**	**0 (0.0%)**	**0**	**0 (0.0%)**

* A1: Vaccine product-related reaction; A2: Vaccine quality defect-related reaction; A3: Immunization error-related reaction; A4: Immunization anxiety-related reaction; B1: Temporal relationship is consistent but there is insufficient definitive evidence that vaccine caused the event; B2: Reviewing factors result in conflicting trends of consistency and inconsistency with causal association to immunization; C: Inconsistent causal association to immunization (coincidental); D: Case without adequate information for causality conclusion.

In all age cohorts within 28 days post first dose, 10 participants (13.9%) in Test and 6 (8.3%) in Comparator groups had acute respiratory infections. Fever was reported by 3 participants (4.2%) in Test and 1 (1.4%) in Comparator groups. Within 28 days post second dose, 10 participants (14.7%) in Test and 12 (17.7%) in Comparator groups had acute respiratory infections. Fever was reported by 4 participants (5.9%) in Test and 2 (2.9%) in Comparator groups.

Five participants (6.9%) in both Test and Comparator groups had elevated liver enzymes post first dose, all asymptomatic. Blood creatinine was mildly elevated in 2 participants (2.8%) in Test and 4 (5.6%) in Comparator groups post first dose. Elevated lab values returned to normal in subsequent follow-up lab tests. All participants were asymptomatic and abnormal lab values were assessed as ‘not clinically significant’.

No SAE was reported throughout the study.

### 3.3 Immunogenicity evaluation

**Fig. 2:**
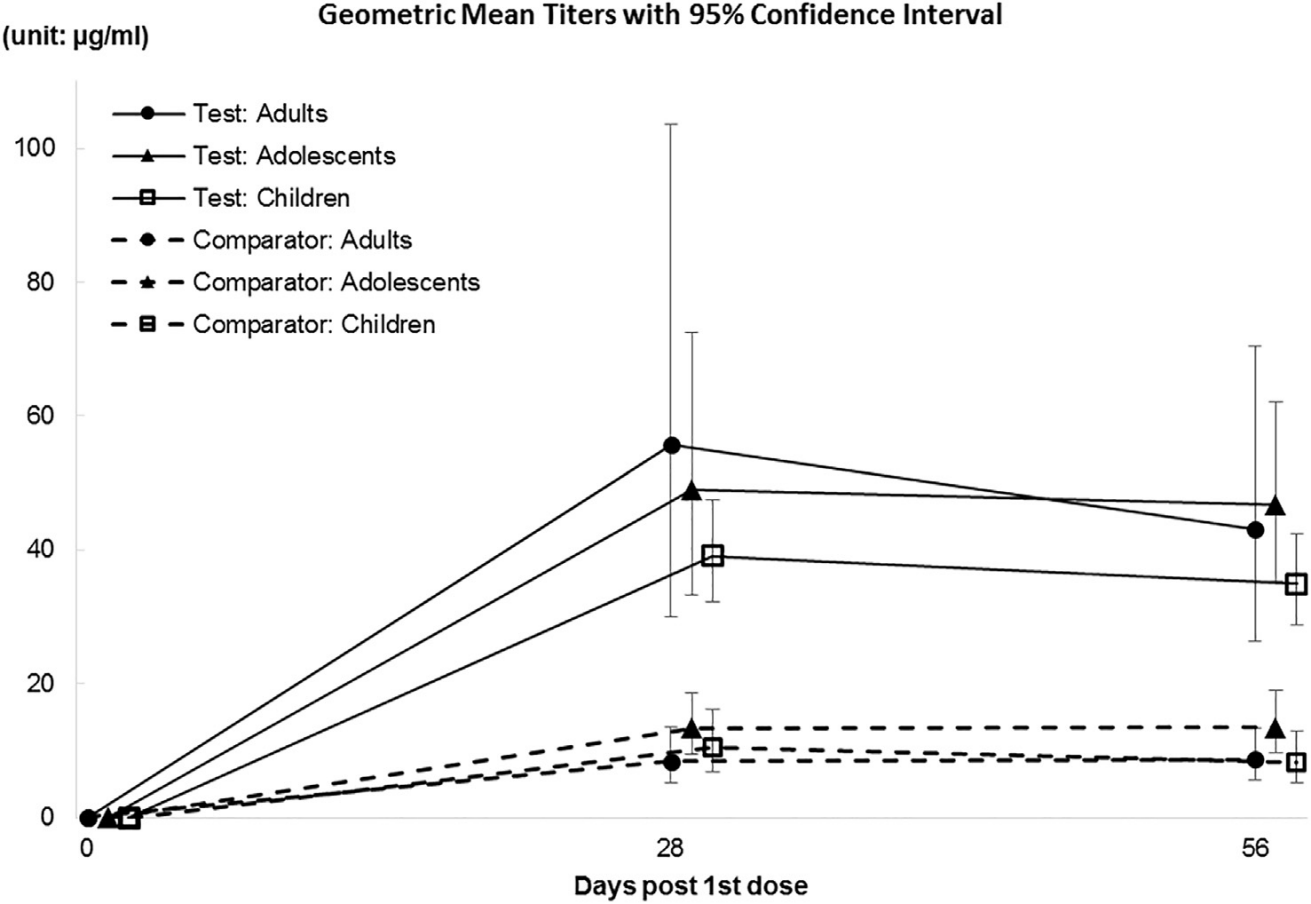
Anti-Vi serum IgG ELISA antibody response (titers in μg/mL) by Age group.

As per immunogenicity set analysis all participants in Test group (100%) showed seroconversion post first and second doses vs 97% in Comparator group. Test group had about a 4-fold higher anti-Vi IgG ELISA GMT than the Comparator group (p < 0.001) (Table 4). Results were similar by age cohort (Fig. 2**).**

**Table 4:** Anti-Vi IgG ELISA response by vaccine groups – immunogenicity set analysis.

All Ages:				
	Time point	Test Group	Comparator Group	P-value†
Number of participants	Day 0	71	72	–
	Day 28	69	69	–
	Day 56	71	72	–

Seroconversion rate^a^ (95% CI)	Day 28	100.0 (94.73, 100.0)	97.10 (90.03, 99.20)	0.143
	Day 56	100.0 (94.87, 100.0)	97.22 (90.43, 99.23)	0.153

GMT^b^ (95% CI)	Day 0	0.02 (0.01, 0.03)	0.02 (0.01, 0.03)	0.771
	Day 28	47.37 (37.34, 60.08)	10.70 (8.50, 13.48)	<0.001
	Day 56	41.42 (34.13, 50.27)	9.88 (7.84, 12.46)	<0.001

GMFrise^c^ (95% CI)	Day 28	2751.62 (1541.73, 4910.97)	511.46 (284.11, 920.73)	<0.001
	Day 56	2430.32 (1385.84, 4262.00)	526.80 (294.40, 942.65)	<0.001

a Proportion of participants who had 4-fold rise in titers compared to baseline (Day 0) to post dose.

b Geometric Mean Titers (unit: µg/ml).

c Geometric Mean Fold rise from baseline (Day 0) to post dose.

† P-values for comparison of Seroconversion rates have been derived using stratified Chi-square (Cochran-Mantel-Haenszel) test stratified by age. P-values for comparison of GMTs or GMF rise was adjusted for age strata in the model using ANCOVA.

Test group seroconversion rates were 100% at day 28 post first dose and post second dose, while GMT decreased from 47.4 µg/mL to 41.4 µg/mL, respectively. Geometric Mean Fold rise from baseline to post first dose was 2751.6 decreasing to 2430.3 post second dose.

SBA seroconversion rates were significantly higher in Test than in Comparator groups post first and second doses (71.0% vs. 52.2%, P-value 0.022 and 70.4% vs. 51.4%, p = 0.019, respectively. SBA GMT were also significantly higher in Test than in Comparator groups post first and second doses (526.6 vs. 271.3, p = 0.016 and 586.5 vs. 223.0, p < 0.001 respectively) (Supplementary Table 1). Results were similar per age cohort.

Anti-DT responses rate for all age groups were 81.2% and 84.5% post first and second Vi-DT doses, respectively, while 4.4% and 5.6% in Comparator group. GMF rise post first and second dose was more than 20 times compared to baseline in Test group while there was no rise in Comparator group (Supplementary Table 2). Results were similar per age cohort.

Overall PP analysis results were in agreement with the immunogenicity set analysis.

## 4 Discussion

Conjugation to a carrier protein converts T cell-independent antigens into T cell-dependent ones, thereby, providing a long lasting immune response by enhancing memory [23]. Carrier proteins such as recombinant exoprotein A from *Pseudomonas aeruginosa* (rEPA), Tetanus toxoid (TT), Diphtheria toxoid (DT) or a non-toxic mutant of diphtheria toxin (CRM 197) have been used with success for Vi polysaccharide conjugate vaccines [24], [25], [26]. Dose-finding studies with Vi-rEPA, using 3 dose levels of 5, 12.5, and 25 µg revealed a dose-dependent increase in anti-Vi IgG responses, with 25 µg eliciting the highest anti-Vi IgG titers [27]. DT is known for its safety profile and considered a reliable carrier protein successfully used for meningococcal conjugate vaccines [28]. Typhoid Vi polysaccharide conjugated to DT as carrier protein was therefore a logical choice for development. We evaluated Vi-DT at the dose of 25 µg based on dose selection done with various typhoid conjugate vaccines in development.

In this Phase I study, Vi-DT showed to be safe, well tolerated, and immunogenic in all age cohorts from 2 to 45 years. No SAE was reported in either Test or Comparator groups. No participant withdrew from the study due to AE. All solicited and unsolicited AEs were mild or moderate in intensity in both vaccine groups with the exception of a 4-year old girl in Test group with grade 3 fever that resolved without sequelae. The overall Vi-DT safety profile is in line with those of other Vi polysaccharide conjugate vaccines [21], [23], [25], [27], [29].

All participants in Test group (100%) showed seroconversion, i.e., 4-fold rise in serum anti-Vi IgG ELISA after first and second doses while the proportion in the Comparator group was 97% post first dose and second dose. Test group showed about 4-fold higher GMT compared to Comparator group. No further increase of GMT was detected post second dose of Vi-DT compared to post first dose, which suggests a single dose may be sufficient to achieve the same level of immune response in 2–45 year age group. The results from this study are in agreement with other studies of typhoid conjugate vaccines in similar age cohorts. One of the studies that tested PedaTyph Vi-TT conjugate vaccine reported 1.8 EU/mL (95% Cl 1.5, 2 EU/mL) as baseline GMT which increased to 32 EU/mL (95% Cl 27, 39 EU/mL) at 6-weeks post first dose [23]. PedaTyph elicited similar levels of anti-Vi IgG in infants and older children (2- to 5-year-old). In adults, PedaTyph was significantly more immunogenic than Vi alone [16]. Typbar-TCV from Bharat biotech was administered as single dose vs. polysaccharide vaccine (Typbar) in children 2–4 and 5–15 years of age, where the conjugate vaccine was significantly more immunogenic, with a 152- and 168-fold rise in GMT at day 42 over baseline, compared to a 46- and 37-fold rise elicited by Typbar, respectively [29]. In a phase I clinical trial of O-acetylated pectin conjugate typhoid vaccine conducted in young children, and a >4-fold rise of anti-Vi IgG GMT was observed 6 weeks post injection [28]. In a Phase I study testing Vi-CRM197 in European adults, anti-Vi IgG GMT levels four weeks post vaccination were six times higher than those vaccinated with Vi alone (304 vs. 52 EU/mL) [24].

Serum bactericidal assays have been widely used to assess immunogenicity of bacterial vaccines such as cholera and meningococcal vaccines due to its correlation with protection [30], [31]. We measured SBA responses to assess whether typhoid vaccines could induce functional *S*. Typhi- specific antibodies after vaccination. SBA titers showed significantly higher GMT in Test vs. Comparator groups, consistent with anti-Vi IgG ELISA results (Supplementary Table 1). In addition, a correlation, although weak, was observed between anti-Vi IgG ELISA and SBA titers in all groups combined (Spearman correlation coefficient 0.282, p < 0.0174) (Supplementary Fig. 1). A similar correlation was observed in a previous study [22] and SBA titers were inversely correlated with susceptibility to typhoid fever [32]. In contrast, natural infection of S. Typhi in an endemic area did not show a correlation between the two types of antibody titers [32]. One possible explanation is that predominant antibody responses are directed to other bacterial components such as LPS or membrane proteins in patients. Vi-DT contains a small amount of endotoxin (9.65 EU/dose, provided by manufacturer) which is within acceptable range for human vaccines. Given the fact that antibody responses to LPS were reported in the Vi polysaccharide vaccination group [33] and depletion of LPS-specific antibody in serum significantly reduced SBA activity, residual LPS of *S.* Typhi in both Vi and Vi-DT vaccines may induce SBA response in both Vi and Vi-DT vaccinees. Some studies suggest that anti-Vi IgG contributed to reduce disease symptoms and prevention of infection in some individuals [34], [35]. However, recently, it was shown that bactericidal antibody reduced typhoid severity but not protection against disease in a human challenge model [36].

Our results show that Vi-DT is safe and immunogenic in 2–45 year old participants and set the stage for further clinical development of Vi-DT in children less than two years of age.
