# Supplementary material for: Safety and immunogenicity of a Vi-DT typhoid conjugate vaccine: Phase I trial in Healthy Filipino adults and children
Source: Vaccine. 2018 Jun 18;36(26):3794–801. doi: 10.1016/j.vaccine.2018.05.038 (PMC6005168; doi:10.1016/j.vaccine.2018.05.038)

**Supplementary Table 1:** **SBA antibody titers by vaccine groups – Immunogenicity Analysis Set**

| **All Ages** |  |  | |  |
| --- | --- | --- | --- | --- |
|  | **Time point** | **Test Group** | **Comparator Group** | **P-value†** |
| Number of participants | Day 0 | 71 | 72 | -- |
|  | Day 28 | 69 | 69 | -- |
|  | Day 56 | 71 | 72 | -- |
| Seroconversion rate^a^ (95% CI) | Day 28 | 71.01 (59.43, 80.38) | 52.17 (40.59, 63.53) | 0.022 |
|  | Day 56 | 70.42 (58.98, 79.77) | 51.39 (40.07, 62.57) | 0.019 |
| GMT^b^ (95% CI) | Day 0 | 95.74 (70.93, 129.24) | 65.39 (47.93, 89.21) | 0.066 |
|  | Day 28 | 526.56 (338.68, 818.68) | 271.26 (184.53, 398.73) | 0.016 |
|  | Day 56 | 586.50 (388.03, 886.50) | 222.97 (157.96, 314.75) | <0.001 |
| GMFrise^c^ (95% CI) | Day 28 | 5.45 (3.52, 8.43) | 3.98 (2.80, 5.66) | 0.253 |
|  | Day 56 | 6.13 (4.06, 9.24) | 3.41 (2.50, 4.64) | 0.024 |

a Proportion of participants who had 4-fold rise in titers compared to baseline (Day 0) to post dose

b Geometric Mean Titers

c Geometric Mean Fold rise from baseline (Day 0) to post dose

† P-values for comparison of Seroconversion rates have been derived using stratified Chi-square (Cochran-Mantel-Haenszel) test stratified by age. P-values for comparison of GMTs or GMFrise was adjusted for age strata in the model

**Supplementary Table 2. Anti-DT antibody response by vaccine groups - Immunogenicity Analysis Set**

| **All Ages** |  |  | |  |
| --- | --- | --- | --- | --- |
|  | Time point | Test Group | Comparator Group | P-value† |
| Number of participants | Day 0 | 71 | 72 | -- |
|  | Day 28 | 69 | 69 | -- |
|  | Day 56 | 71 | 72 | -- |
| Seroconversion rate^a^ (95% CI) | Day 28 | 81.16 (70.39, 88.65) | 4.35 (1.49, 12.02) | <0.001 |
|  | Day 56 | 84.51 (74.35, 91.12) | 5.56 (2.18, 13.43) | <0.001 |
| GMT^b^ (95% CI) | Day 0 | 0.08 (0.05, 0.13) | 0.11 (0.07, 0.17) | 0.309 |
|  | Day 28 | 2.20 (1.25, 3.86) | 0.11 (0.07, 0.17) | <0.001 |
|  | Day 56 | 1.93 (1.13, 3.27) | 0.11 (0.07, 0.17) | <0.001 |
| GMFrise^c^ (95% CI) | Day 28 | 26.30 (16.30, 42.44) | 0.93 (0.79, 1.10) | <0.001 |
|  | Day 56 | 23.11 (14.70, 36.33) | 0.97 (0.81, 1.15) | <0.001 |

a Proportion of participants who had 4-fold rise in titers compared to baseline (Day 0) to post dose

b Geometric Mean Titers (unit: IU/ml)

c Geometric Mean Fold rise from baseline (Day 0) to post dose

† P-values for comparison of Seroconversion rates have been derived using stratified Chi-square (Cochran-Mantel-Haenszel) test stratified by age. P-values for comparison of GMTs or GMFrise was adjusted for age strata in the model

**Supplementary Figure 1: Correlation between Anti-Vi IgG ELISA and SBA titers**


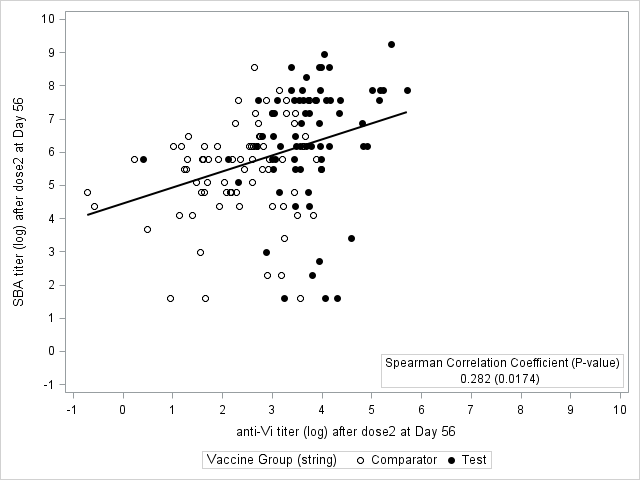

Supplement: Supplementary data 1 [file mmc1.docx]
